# MRI of odontogenic maxillofacial infections: diagnostic accuracy and reliability

**DOI:** 10.1007/s11282-022-00646-7

**Published:** 2022-08-09

**Authors:** Jaakko Heikkinen, Viljami Jokihaka, Janne Nurminen, Ville Jussila, Jarno Velhonoja, Heikki Irjala, Tero Soukka, Tatu Happonen, Jorma Järnstedt, Mikko Nyman, Kimmo Mattila, Jussi Hirvonen

**Affiliations:** 1 Department of Radiology, University of Turku and Turku University Hospital, Kiinamyllynkatu 4-8, 20520 Turku, Finland; 2 Department of Otorhinolaryngology-Head and Neck Surgery, University of Turku and Turku University Hospital, Turku, Finland; 3 Department of Oral and Maxillofacial Surgery, University of Turku, Turku, Finland; 4 Medical Imaging Center, Department of Radiology, Tampere University and Tampere University Hospital, Tampere, Finland

**Keywords:** Magnetic resonance imaging, Odontogenic abscess, Emergency medicine, Infection

## Abstract

**Objectives:**

To determine the diagnostic accuracy of emergency magnetic resonance imaging (MRI) in odontogenic maxillofacial infections, the clinical and surgical significance of MRI findings, and whether MRI can identify the tooth responsible for the infection.

**Methods:**

A retrospective cohort study reviewed 106 emergency neck MRI scans of patients with neck infections of odontogenic origin. The diagnostic accuracy of MRI in identifying abscesses was studied relative to surgical findings. Correlations were analyzed between various MRI findings and clinical results and outcomes, such as the surgical approach (intraoral *vs.* extraoral). The ability of MRI findings to predict the causative tooth was assessed in a blinded multi-reader setting.

**Results:**

Of the 106 patients with odontogenic infections, 77 (73%) had one or more abscesses. Imaging showed a sensitivity, specificity, and accuracy of 0.95, 0.84, and 0.92, respectively, for MRI diagnosis of an odontogenic abscess. Among the imaging findings, mediastinal edema was the strongest predictor of extraoral surgery. MRI showed bone marrow edema in the majority of patients, and multi-reader assessment showed good reliability. MRI was also able to predict the causative tooth accurately.

**Conclusions:**

Emergency neck MRI can accurately detect odontogenic abscesses and reliably point to the causative tooth. These results can increase the utility and reliance on emergency MRI in clinical decision-making.

**Supplementary Information:**

The online version contains supplementary material available at 10.1007/s11282-022-00646-7.

## Introduction

The incidence of oral infections is rising, and they are currently the most common cause of deep neck infections that require hospitalization [1, 2]. Advanced infections in these patients can lead to life-threatening conditions, including airway compromises, vascular complications, septicemia, and mediastinitis. In addition, clinical factors such as older age, smoking, diabetes, multi-space infection, and the demonstration of a causative molar tooth have been associated with a more extended hospital stay among patients with odontogenic infections [3]. Odontogenic infections have well-established management protocols involving antimicrobial drugs and incision and drainage of abscesses. Death from odontogenic infections is rare and is often associated with prolonged hospital care/mechanical ventilation and secondary pneumonia [4]; early diagnosis is the key to preventing severe complications.

In clinical practice, determining whether the symptoms and findings reflect tissue cellulitis or phlegmon rather than actual abscess formation can be challenging. Emergency imaging is often required in severe cases to determine the exact diagnosis and extent of the abscess in deep neck infections and for accurate and timely intervention [5, 6]. Multidetector computed tomography (CT) of the neck soft tissues or cone-beam computed tomography (CBCT) of bony structures is considered the standard imaging method for acute odontogenic infections [6, 7]. Although MRI is considered time-consuming and challenging for acutely ill patients, emergency neck MRI has been shown to be feasible and to have better diagnostic accuracy than that previously reported for CT [8]. In a prospective head-to-head comparison, MRI was also considered superior to CT in the initial evaluation of acute neck infections [9, 10]. MRI can also reliably detect abscesses [8] and visualize specific edema patterns associated with more severe illnesses [11, 12]. Despite the growing incidence of odontogenic infections and the increasing awareness of the utility of emergency MRI imaging, detailed MRI imaging patterns in serious odontogenic infections and their clinical significance, as well as how accurately MRI can pinpoint the causative tooth, are unknown.

This retrospective cohort study aimed to characterize MRI findings in clinically confirmed odontogenic infections. The purposes of this study were to assess the diagnostic accuracy of MRI in odontogenic abscess detection relative to surgical findings as a reference standard, to study whether MRI findings can predict the extraoral surgical approach and to examine whether MRI can accurately identify the causative tooth.

## Materials and methods

The authors obtained permission from the hospital district board for this retrospective cohort study in a single academic tertiary care referral center. Reviews were not sought from the institutional review board (IRB) (approval or waiver) because the national legislature does not require this for retrospective studies of existing data. The inclusion criteria were as follows: (1) emergency MRI for suspected neck infection between April 1, 2013, and December 31, 2018; (2) MRI evidence of infection: high signal of fat-suppressed T2-weighted Dixon images suggesting edema, or high signal of fat-suppressed post-Gadolinium (post-Gd) T1-weighted Dixon images suggesting abnormal tissue enhancement; (3) final clinical diagnosis of odontogenic infection in the medical records (as reference standard); and (4) diagnostic image quality deemed by the radiologist reading the study. Using standard neck MRI codes, cases were identified using picture archiving and communication systems (PACS) and radiological information systems (RIS), and the data were cross-referenced with patient medical files.

MRI was performed with a Philips Ingenia 3 Tesla system using a dS HeadNeckSpine coil configuration (Philips Healthcare) and Gd-based contrast agent (Dotarem^®^, Guerbet). The supplemental material provides details of the MRI protocol (approximately 30 min). All MRI data were retrospectively reviewed by two fellowship-trained neuroradiologists (J.Hi. and J.He.). MRI criteria for infection and abscess and surgical methods for abscess detection have been previously described [8, 11, 12]. Both the detection [8] and measurement [11] of an abscess have substantial interobserver reliability. For accuracy of abscess detection, the surgical records were reviewed for the presence of pus and were considered the reference standard for abscess diagnosis.

Abscess size (maximal diameter) and edema patterns were evaluated to study whether MRI findings predict the surgical approach (intraoral vs. extraoral). On the basis of the fat-suppressed T2-weighted Dixon images, the authors evaluated edema as a high signal in the submandibular space (SMS; inferior to the mylohyoid muscle), sublingual space (SLS; superior to the mylohyoid muscle), visceral space (VS; infrahyoid soft tissue space including the larynx, surrounding strap muscles, and the thyroid; also including edema in the anterior cervical space between the sternocleidomastoid muscle and the carotid space [12]), retropharyngeal space edema (RPE), and mediastinal edema (ME) [11]. These edema patterns have a substantial interobserver agreement [11, 12]. Surgical records were retrospectively reviewed to determine whether the surgical approach was intraoral or extraoral.

To study whether MRI can accurately demonstrate the causative tooth, three board-certified and fellowship-trained specialist radiologists (M.N., a neuroradiologist with 15 years of experience; V.J., an emergency radiologist with ten years of experience; and J.N., a musculoskeletal radiologist with nine years of experience) independently evaluated a subsample of patients without a previous dental procedure, in whom the causative tooth was unknown at the time of imaging but could later be reliably inferred from clinical notes (clinical reference standard). All readings were blinded to other imaging data, ratings from other radiologists, and clinical data, including referrals. Radiologists were asked to rate the presence or absence of bone marrow changes (in T1-weighted, fat-suppressed T2-weighted, fat-suppressed contrast-enhanced T1-weighted, and DW images), cortical erosions, periapical abnormalities, and artifacts. They were also asked to propose the causative tooth, for their opinion on the most useful MRI sequence to evaluate this and to rate the subjective level of confidence of this assessment on a scale of 1 to 5.

The results are expressed as percentages, means, and standard deviations (SDs). The authors used independent samples T-tests to compare continuous variables and Chi-square (*Χ*^2^) tests to compare ordinal data. The sensitivity, specificity, and accuracy of MRI *vs*. surgery were calculated as previously described [8]. For the multivariate prediction model for extraoral surgery, a binary logistic regression model with two methods of variable selection was used: enter all and forward stepwise. In the multi-reader study on the causative tooth, percentage agreement was calculated for both accuracy (MRI *vs.* clinical reference standard) and interobserver agreement. The Fleiss multi-reader Kappa was also used to assess the agreement on the causative tooth. Data were analyzed using IBM SPSS Statistics for Mac (version 26, copyright IBM Corporation 2019). P-values less than 0.05 were considered statistically significant.

## Results

### Diagnostic accuracy of MRI

**Fig. 1:**
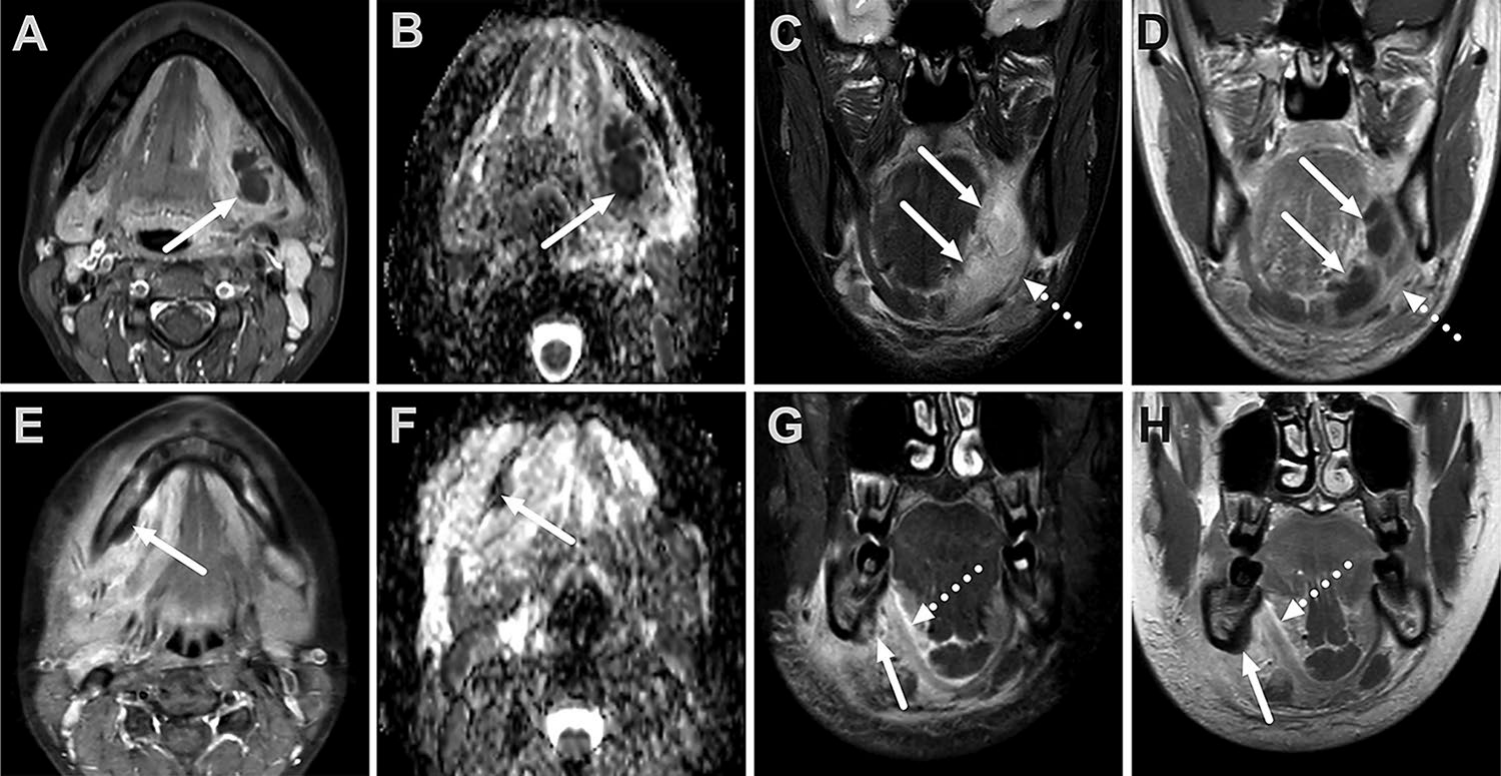
Sublingual space abscess (solid arrows) in a 25-year-old female following teeth extraction (dd. 28,38) and subsequent infection (upper row). Small submandibular space abscess (solid arrows) subperiosteally in a 13-year-old male with d47 periapical infection (lower row). T1 fat-suppressed axial images after gadolinium (**A** and **E**), showing the nonenhancing abscess core surrounded by an enhancing rim. The abscess shows restricted diffusion in the ADC map (**B** and **F**). Coronal T2 fat-suppressed and T1 post-contrast Dixon images show the hyperintense and nonenhancing abscess and its relation to the mylohyoid muscle (dotted arrows) in the sublingual space (**C** and **D**) and submandibular space (**G** and **H**)

**Fig. 2:**
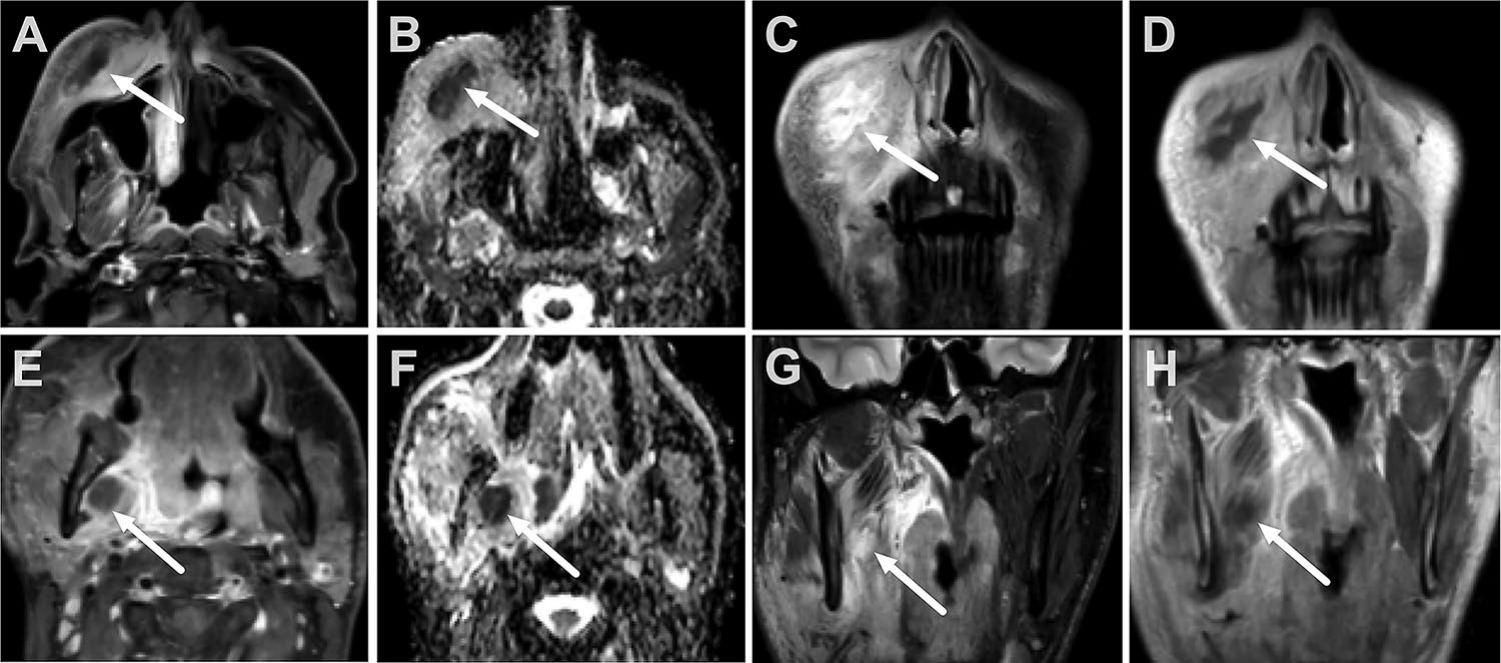
Buccal space abscess in a 32-year-old male with recent root canal treatment (upper row). Masticator space abscess in a 30-year-old male with odontogenic infection after teeth extraction (dd37,38) (lower row). T1 fat-suppressed axial images with gadolinium (**A** and **E**) show the nonenhancing abscess core surrounded by an enhancing rim. The abscess shows restricted diffusion in the ADC map (**B** and **F**). Coronal T2 fat-suppressed Dixon images show the hyperintense abscess and surrounding edema (**C** and **G**). T1 coronal Dixon in-phase images with gadolinium show the nonenhancing abscess (**D** and **H**)

The study consisted of 106 patients (67 male, 39 female) with a clinically confirmed acute odontogenic infection who underwent MRI imaging in the emergency department (Table 1). Imaging showed evidence of one or more abscesses in 81 patients (76%), of whom 73 (90%) underwent surgery (Table 2). This surgically managed group had four false positives and 69 true positives. In addition, 14 (56%) patients out of the 25 with no MRI evidence of an abscess underwent a surgical operation, and pus was found in four cases (false negatives). These results led to sensitivity, specificity, and accuracy of 0.95, 0.84, and 0.92, respectively, for MRI diagnosis of an abscess. The positive and negative predictive values were 0.95 and 0.84, respectively. The most common abscess location was submandibular (49%), followed by the sublingual space (18%) (Figs. 1 and 2). Of the 87 total surgical interventions, 52 (60%) were performed intraorally, and 35 (40%) required extraoral surgery. Patients with multi-space abscesses, parapharyngeal extension, and no previous dental procedure had the most severe course of illness (details provided in Supplementary Data Table 6).

**Table 1:** Patient characteristics

Characteristic	
Number of patients	106
Age (years, mean ± SD)	43 ± 17
Male (N, %)	67 (63%)
Female (N, %)	39 (37%)
BMI (kg/m^2^)	28.4
CRP (mg/L, mean ± SD)	126^a^ ± 88
WBC (× 10^9^/L, mean ± SD)	14.0^a^ ± 5.8
Body temperature (℃, mean ± SD)	37.5^b^ ± 0.7
Duration of symptoms before imaging (days, mean ± SD)	4.0^c^ ± 3.5
ICU	12 (11%)
Length of hospital stay (LOS) (days, mean ± SD)	4.0^c^ ± 3.5
Prior procedure	79 (75%)
Surgery	87 (82%)
-Extraoral	35 (40%)
-Intraoral	52 (60%)

Data available for ^a^102, ^b^77, and ^c^103 patients

**Table 2:** Imaging characteristics. Values are *N* (%) or mean ± SD

Outcome	
Abscess	77 (73%)^a^
Maximal abscess diameter (mm, mean ± SD)	35 ± 17
Abscess location (primary)	
Submandibular	38 (49%)
Sublingual	14 (18%)
Buccal	11 (14%)
Masticator	10 (13%)
Parapharyngeal	4 (5%)
Multiple	29 (38%)
Subperiosteal	22 (27%)
Edema, presence	
Submandibular (SMS)	102 (96%)
Sublingual (SLS)	82 (77%)
Visceral (VS)	81 (76%)
All (SMS + SLS + VS)	69 (65%)
Retropharyngeal (RPE)	37 (35%)
Mediastinal (ME)	20 (23%)
Bone signal changes	
Low T1	59 (56%)
High T2	83 (78%)
Enhancement	80 (76%)
Diffusion restriction	2 (2%)

^a^true positives

### MRI predictors of surgical approach

Patients who underwent extraoral surgery *vs.* intraoral operation had a higher CRP (168 *vs*. 111 *p* = 0.003), higher WBC (15.3 *vs*. 12.5, *p* = 0.008), larger abscesses (41 mm *vs*. 32 mm *p* = 0.024), and a higher prevalence of VS and SLS edema (for both, 89% *vs.* 69% *p* = 0.041), ME (43% *vs*. 8.9% *p* = 0.001) and RPE (51% *vs.* 25% *p* = 0.022). None of these significant univariate variables could predict an extraoral surgical approach in the multivariate analysis when all the variables were forced into the model simultaneously, but in the stepwise model, ME predicted extraoral surgery (*p* = 0.002) (Table 3).

**Table 3:** Univariate and multivariate modeling of the extraoral surgical approach

Univariate			Multivariate (enter)		Multivariate (stepwise)	
	Statistic*	*p*-value	Odds ratio	*p*-value	Odds ratio	*p*-value
Age	− 0.267	0.790				
CRP	− 3.091	**0.003**	1.005	0.381	2.365	0.124
WBC	− 2.697	**0.008**	1.097	0.281	3.126	0.077
Location	7.077	0.132				
Abscess max. diameter	− 2.309	**0.024**	0.998	0.942	1.317	0.251
SMS	2.091	0.270				
SLS	4.420	**0.041**	3.726	0.173	2.498	0.114
VS	4.420	**0.041**	1.165	0.880	0.744	0.388
ME	11.637	**0.001**	4.412	0.074	8.800	**0.002**
RPE	6.371	**0.022**	0.902	0.887	0.991	0.319
Multi- space	2.324	0.146				
Pre-treatment	5.539	**0.026**	0.379	0.189	2.052	0.152

^*^T value for continuous variables, Chi-square for nominal variables

### Detection of causative tooth

In an analysis of 23 patients with odontogenic infections without a recent dental procedure, the radiologists located the same causative tooth (all mandibular molars except one maxillary molar) in 96% (at least one radiologist), 83% (at least two radiologists), and 57% (all three radiologists) of the cases (Table 4) with moderately high confidence (Supplementary Table 7). The multi-reader Kappa was 0.66, indicating substantial agreement. In 96% of the cases (22/23), the difference between the radiologist and the true causative tooth was one tooth, and in only one case, there was a difference of two teeth between the radiologists and the final diagnosis. In 96% of the cases, at least two of the three radiologists agreed with each other on the causative tooth, and in 61% of the cases, all three radiologists agreed with each other on the infection focus.

**Table 4:** Degree of agreement in radiologists’ assessments of MRI focus teeth compared to the reference standard and dental panoramic radiography

Radiologists agreeing	Interobserver	MRI vs. Reference standard
at least 1 of 3	N.A	96%
at least 2 of 3	96%	83%
3 of 3	61%	57%

MRI, Magnetic Resonance Image. N.A., Not applicable

**Fig. 3:**
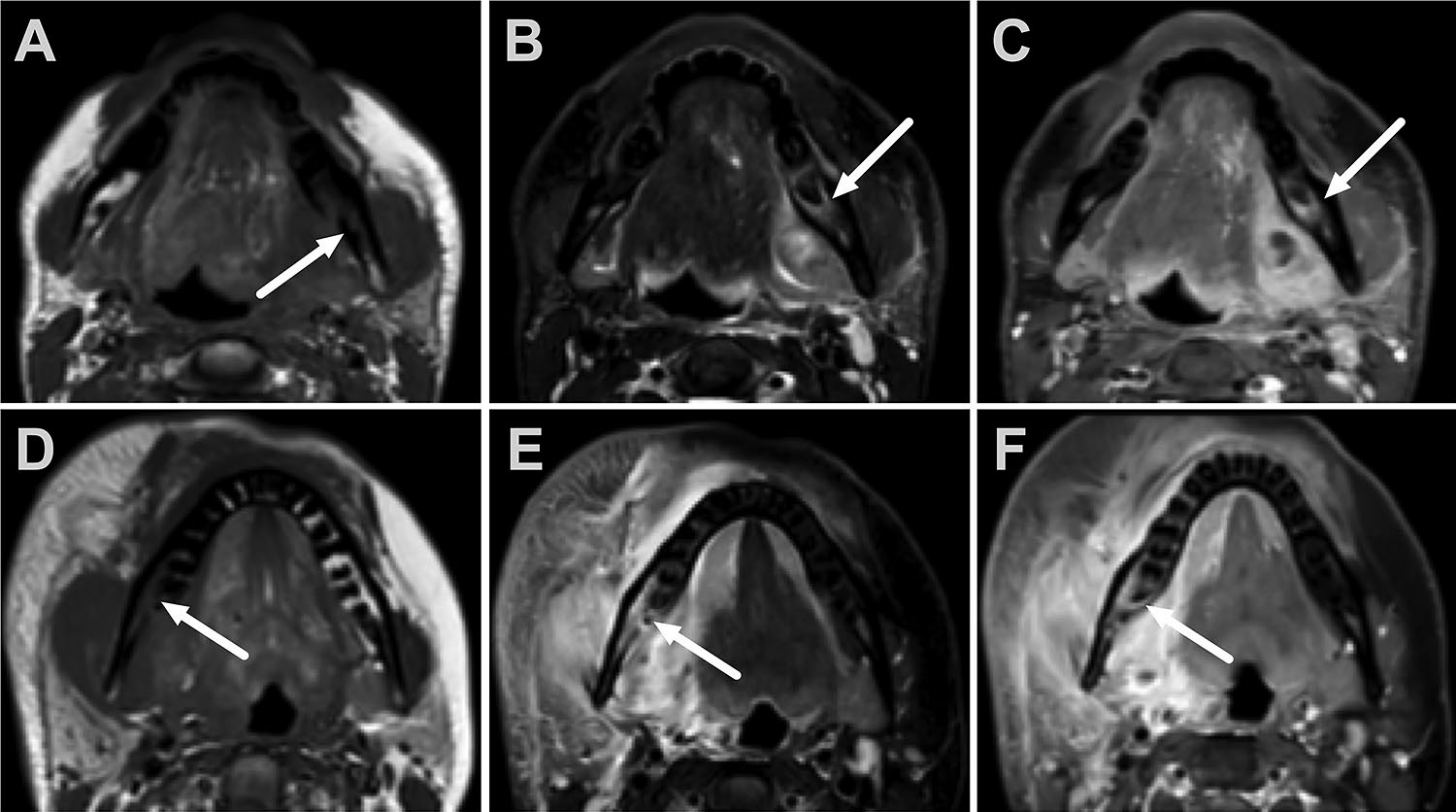
Examples of bone marrow signal changes. Upper row: A 31-year-old male with odontogenic infection and abscess showing low T1 (**A**), high T2 signal (**B**), and enhancement (**C**) in the left mandibular molar region. Lower row: 32-year-old female with odontogenic infection and submandibular abscess (not shown), right-sided molar region bone marrow changes, low T1 (**D**), high T2 (**E**), and enhancement (**E**)

**Fig. 4:**
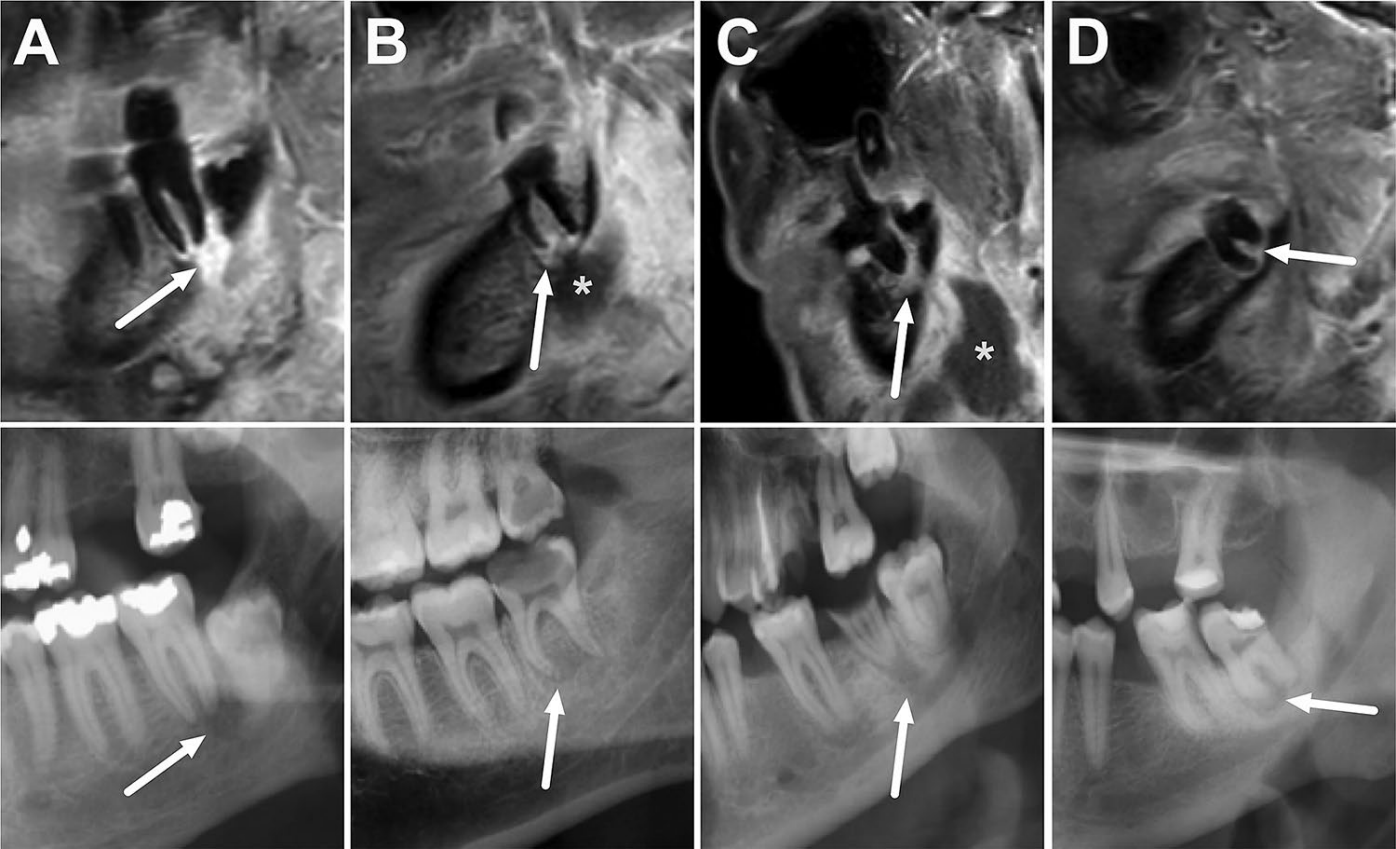
Four patients with odontogenic deep neck infection, periapical infection on T1 fat-suppressed sagittal images with gadolinium (upper row), and corresponding X-ray findings (lower row). Arrows indicate enhancement around the roots (**A** d47; **B** d48; **C** d37; **D** d38) and periapical lucency on X-ray. Asterisks in (**B**) and (**C**) demonstrate adjacent extraosseous abscesses. X-ray images from patients (**A**) and (**B**) have been flipped left–right for visualization purposes

The radiologists preferred contrast-enhanced fat-suppressed T1-weighted images over fat-suppressed T2-weighted and diffusion-weighted images in tooth identification, and bone marrow signal changes were observed with a high agreement (Figs. 3 and 4, Table 5). The overall prevalence of reported artifacts was 10% across all the image readings, but these were mostly considered negligible or mild.

**Table 5:** Radiological bone marrow features related to MRI-identified focus tooth

Imaging feature	Prevalence				
	Rad 1 (%)	Rad 2 (%)	Rad 3 (%)	Overall (%)	Inter-observer agreement (3 of 3 rad) (%)
Preference of T1W FS Gd over T2W FS	74	78	91	81	78
Low signal on T1W	78	65	91	78	65
High signal on T2W FS	100	91	100	97	91
High signal on T1W FS Gd	95	82	95	91	83
Restricted diffusion (DWI)	17	4	22	14	70
Cortical erosion	65	43	52	54	48
Periapical abnormality	91	83	96	90	83

*MRI* Magnetic Resonance Image, *Rad* Radiologist, *T1W* T1-weighted. *FS* Fat saturation, *Gd* gadolinium, *T2W* T2-weighted, *DWI* diffusion-weighted imaging

## Discussion

Previous studies have shown that MRI is a feasible imaging method for diagnosing deep neck infections in an emergency care setting [8] and that MRI has higher diagnostic accuracy for maxillofacial infections than CT [10]. We found that MRI has high diagnostic accuracy for odontogenic abscesses, that MRI findings can predict clinical severity and surgical approach and that MRI can point to the causative tooth. Together, these results add to the growing knowledge on the utility of emergency MRI in acute neck infections.

Accurate delineation of odontogenic abscesses is important for choosing the optimal type and extent of treatment (*e.g.*, surgery). Sensitivity, specificity, and accuracy values of 0.95, 0.84, and 0.92 were found, respectively, for the MRI diagnosis of an odontogenic abscess. The high diagnostic accuracy is consistent with prior reports in larger samples of various types of neck infections [8]. The PPV of 0.95 is also markedly higher than that previously reported for CT (approximately 0.80) [13, 14]. All the false positives (four) were small abscesses (7–22 mm) and had imaging characteristics of an abscess, but no pus had been identified in surgery. However, small abscesses can also be missed in surgery, and pus can emerge between MRI and surgery. These issues may explain some of the false MRI findings in this study.

As expected, extensive infection findings (*e.g.*, parapharyngeal or multi-space abscess) on MRI were associated with a more severe course of the disease (Supplementary Data Table 6). Previously, ME, RPE, and abscess diameter have been shown to predict more severe illnesses in deep neck infection patients with multiple etiologies [11]. Here, ME was a significant predictor of extraoral surgery in patients with odontogenic infections. SLS edema was more common here in odontogenic infections (77%) than was previously shown for tonsillar infections (24%) [12]. In contrast, RPE is less common in odontogenic than in tonsillar infections [11]. Thus, different etiologies of neck infection are associated with distinct soft tissue edema patterns, each with its own clinical significance.

Bone marrow signal changes were present in almost all cases. Based on recent consensus on the nomenclature for MRI of musculoskeletal infection outside the spine, these changes are consistent with acute osteomyelitis [15], although they do not necessarily indicate bone destruction, sequestration, or pus formation inside the bone, as is often associated with this term in the jaw [16]. In patients with suspected deep neck infection or abscess, these bone signal changes may be a valuable indicator of the odontogenic origin of infection because MRI with fat-saturated sequences is very sensitive to bone marrow edema. However, this analysis was restricted to patients already known to have an odontogenic infection, and the authors did not study the differentiation between odontogenic and non-odontogenic infections.

CT and CBCT imaging are considered reference standards for assessing dental emergencies, mostly due to their widespread availability and ability to depict bony structures [6, 7, 17, 18]. Although MRI is considered superior for evaluating soft tissues in deep neck infections [8, 9], whether it can also demonstrate the causative tooth in odontogenic infections has been unclear. Although there was variability among agreement between raters (kappa 0.66), it was found that MRI can pinpoint the causative infected tooth (within a margin of error of one tooth) with good accuracy. The fat-suppressed, contrast-enhanced, T1-weighted sequence was considered the most useful in this regard, and periapical enhancement is particularly suggestive of significant infection (Fig. 4). Gd is recommended for use in odontogenic infections, like in other soft-tissue or musculoskeletal infections [15]. A potential clinical implication of this study is that MRI alone may be sufficient to detect the odontogenic origin of severe neck infections needing medical imaging for suspected abscess, but lack of direct comparison with CT precludes strong practical recommendations. In addition, longer scanning times compared with X-ray or CT may be an issue in clinical practice when imaging the causative tooth.

Artifacts from dental hardware are a common limitation of CT in evaluating the oral cavity [19]. In general, MRI can also suffer from artifacts (such as distortion and signal loss), but these are considered less significant than those of CT in dental imaging [20]. In the subcohort, artifacts from dental hardware were rare and not considered significant in the decision-making.

The strengths of this study are its large sample size, high-quality 3 Tesla MR imaging with a Gd-based contrast agent and DWI, systematic neuroradiological evaluation of MRI findings, blinded multi-reader assessment thorough clinical characterization, and surgical confirmation of abscesses. However, as the study was retrospective in nature, the medical and surgical records may have been incomplete or imprecise. As indications for imaging may vary, the current results may be biased. Circumferential reasoning may also have biased the investigation. The MRI images might have had an impact on the final clinical decision regarding the causative tooth in this study. However, the authors did not consider this likely to significantly bias our results because, in clinical practice, MRI is not considered an established imaging method for indicating the causative tooth in odontogenic infections, and the exact tooth was not usually mentioned in the original reports. Interobserver agreement on the causative teeth was not perfect (Kappa of 0.66 indicates substantial agreement) but much improved when disagreement of one tooth was allowed. While this may indicate difficulties for radiologists in accurately numbering individual teeth (*e.g.*, when some teeth are missing), MRI can pinpoint the region of the infected tooth with acceptable precision. A further limitation of this analysis is that only a subset of patients was included in whom the causative tooth was not known. The surgical results were also sometimes unclear, although in very few cases. The interpretation of some of the MRI findings may be subjective, although a substantial percent agreement was found for the bone marrow signal changes, as has also been found for soft tissue edema patterns [11, 12] and detection of abscesses [8]. The most important limitation is the lack of head-to-head comparisons between MRI and CT, although such data already exist in the literature [10]. The evidence seems to be accumulating that MRI can more accurately show the extent and origin of infection and abscess formation [8, 11, 12]. It should be recognized that MRI may not always be suitable or available for all patients, so these results may not apply to all facilities.

In conclusion, MRI provided clinically meaningful information in patients with odontogenic infections. These results add to our understanding of the clinical utility of MRI in acute neck infections.

## Supplementary Information

Below is the link to the electronic supplementary material.

Supplementary file1 (DOCX 216 KB)

## Abbreviations

ICU: Intensive care unit
LOS: Length of hospital stay
SMS: Submandibular space
SLS: Sublingual space
VS: Visceral space
RPE: Retropharyngeal edema
ME: Mediastinal edema
MRI: Magnetic resonance imaging
CT: Computed tomography
CBCT: Cone-beam computed tomography
CRP: C-reactive protein
WBC: White blood cell count
BMI: Body mass index
