# Supplementary material for: MRI of odontogenic maxillofacial infections: diagnostic accuracy and reliability
Source: Oral Radiol. 2022 Aug 9;39(2):364–71. doi: 10.1007/s11282-022-00646-7 (PMC10017569; doi:10.1007/s11282-022-00646-7)
Supplement: Supplementary file 1 — Supplementary file1 (DOCX 216 KB) [file 11282_2022_646_MOESM1_ESM.docx]

**Supplemental Material**

*Detailed MRI protocol*

MRI was performed on a Philips Ingenia 3 Tesla system using dS HeadNeckSpine coil configuration (Philips Healthcare, Best, Netherlands).

In the majority of the cases (96 of 106, 91%), we used a novel protocol:

| Sequence | Orientation | Parameters |
| --- | --- | --- |
| T1 TSE | axial | slice thickness 4 mm, TE=10 ms, TR=641 ms |
| T2 TSE Dixon | axial | slice thickness 4 mm, TE=100 ms, TR=3021 ms |
| T2 TSE Dixon | coronal | slice thickness 3.5 mm, TE=80 ms, TR=3210 ms |
| DWI | axial | slice thickness 4 mm, TE=87 ms, TR=3981 ms, b-value 1000 s/mm^2^ |
| T1 TSE Dixon after Gd | axial | slice thickness 4 mm, TE=7 ms, TR=634 ms |
| T1 TSE Dixon after Gd | coronal | slice thickness 3.5 mm, TE=14 ms, TR=560 ms |
| T1 TSE Dixon after Gd | sagittal | slice thickness 3 mm, TE=14 ms, TR=630 ms |

In 10 of the 106 cases (9%), we used an older protocol:

| Sequence | Orientation | Parameters |
| --- | --- | --- |
| T2 TSE | axial | slice thickness 3 mm, TE=80 ms, TR=3203 ms |
| T2 SPAIR | coronal | slice thickness 3 mm, TE=80 ms, TR=3608 ms |
| T1 TSE | sagittal | slice thickness 3 mm, TE=16 ms, TR=641 ms |
| DWI | axial | slice thickness 4 mm, TE=86 ms, TR=4843 ms, b-value 1000 s/mm^2^ |
| T1 SPIR after Gd | axial | slice thickness 3 mm, TE=18 ms, TR=651 ms |
| T1 TSE after Gd | coronal | slice thickness 3 mm, TE=16 ms, TR=604 ms |

**Table 6** Effects of previous procedures, parapharyngeal and multi-space abscess

| Previous dental treatment | 1 | 0 | p-value |
| --- | --- | --- | --- |
| WBC | 12.6 x 10^9^/L | 16.6 x 10^9^/L | 0.002 |
| Abscess size | 31 mm | 42 mm | 0.008 |
| Extraoral surgery | 33% | 61% | 0.026 |
| LOS | 3.3 days | 5.1 days | 0.069 |

| Parapharyngeal abscess | 1 | 0 | p |
| --- | --- | --- | --- |
| CRP | 263 mg/l | 134 mg/l | 0.019 |
| LOS | 11.0 day | 4.4 days | 0.001 |
| ICU | 75% | 25% | 0.011 |

| Multi-space abscess | 1 | 0 | p |
| --- | --- | --- | --- |
| CRP | 173 mg/l | 110 mg/l | 0.002 |
| WBC | 15.8 | 12.8 | 0.037 |
| Abscess size | 43 mm | 30 mm | 0.001 |
| LOS | 6.0 days | 3.4 days | 0.004 |
| ME | 40% | 17% | 0.039 |
| RPE | 59% | 29% | 0.011 |
| ICU | 28% | 8.3% | 0.024 |

**Table 7** Confidence of focus tooth identification

|  | Rad 1 | Rad 2 | Rad 3 | Overall |
| --- | --- | --- | --- | --- |
| Confidence of focus tooth identification on scale of 1–5 | 3.3 (1.1) | 4.4 (0.7) | 4.0 (0.8) | 3.9 (1.0) |

MRI, Magnetic Resonance Image. DPR, Dental Panoramic Radiography. N.A., Not Applicable. Values are mean (standard deviation)
